# Apps for Health-Related Education in Pharmacy Practice: Needs Assessment Survey Among Patients Within a Large Metropolitan Area

**DOI:** 10.2196/resprot.5886

**Published:** 2017-07-19

**Authors:** Sean M Mirk, Nicole Marie Wegrzyn

**Affiliations:** ^1^ Department of Pharmacy Practice Chicago College of Pharmacy Midwestern University Downers Grove, IL United States; ^2^ Department of Pharmacy Practice Albany College of Pharmacy and Health Sciences Albany, NY United States; ^3^ CareMore Health Plan Tucson, AZ United States

**Keywords:** patient education, mHealth, health information management, user-centered design

## Abstract

**Background:**

Patient education resources are crucial to the effectiveness of prescribed pharmacotherapy. However, user interest and patient preference for these materials is lacking. Regardless of the field, nearly every article on designing mHealth apps references the lack of end-user involvement as a key flaw to sustainable design. The traditional paper-based methods of patient education are difficult to tailor to a patient’s specific needs and learning styles, but a customizable app might be beneficial.

**Objective:**

Regarding a mobile app for patient education, the objectives of the study were to (1) quantify patient interest, (2) determine desirable features, and (3) determine if a relationship exists between patient variables and interest in an iPad app for patient education.

**Methods:**

A paper-based questionnaire was developed and administered to consenting patients receiving care within three sites: two suburban outpatient sites where ambulatory care services are provided and one urban hospital site where ambulatory care transition services are provided.

**Results:**

A total of 121 surveys were completed. Most respondents were female (64/120, 53.3%), between 50 and 70 years of age, white/Caucasian (94/120, 78.3%), owned at least one technology device, and knew what an iPad was. Diabetes was the most common disease state (43/120, 35.8%), followed by heart failure (27/120, 22.5%), history of venous thromboembolism (VTE) (21/120, 17.5%), and asthma/chronic obstructive pulmonary disease (17/120, 14.2%). Overall interest in a mobile health app for patient education was 63.7% (72/113). Interest increased to 68.4% (78/114) if a health care provider recommended it. Respondents with one chronic health condition were more likely to be interested in an app compared to those with two or more. Respondents with a history of VTE were mostly likely to be interested in using an app on their own accord, while respondents with diabetes were mostly likely to be interested if their health care provider recommended it.

**Conclusions:**

This preliminary needs assessment identified that patients are interested in using mHealth apps for health-related education in pharmacy practice, particularly if their health care provider recommends it.

## Introduction

Mobile devices are growing increasingly popular. Based on recent data from Pew Internet Research surveys, 32% of adults in the United States own an e-reader, 42% own a tablet, and 64% own a mobile phone [1-2]. In the past year, a large majority (62%) of mobile phone users have used their phones to look up health information. Furthermore, a larger number of individuals access the Internet from a mobile device than from a traditional computer [3].

Earlier studies have examined the use of personal digital assistants (PDAs) for health care professionals and patients. Most studies utilized PDAs for journaling or data collection [4-6]. These early studies demonstrate the usefulness of a mobile device to support daily clinical activities and as a preferred method for diary entries by patients. Mobile devices today (eg, mobile phones and tablets) have advanced capabilities and provide greater functionality; this has created a boon in mobile health apps.

The latest published information shows there are around 165,000 mobile health apps available in major app stores [7]. Patient-centered mobile health apps target major disease states (eg, diabetes, asthma, heart failure, and chronic pulmonary disorders), promote wellness and healthy habits, provide information and education, allow for tracking of health information, promote engagement with health care providers, and leverage social influence [8,9]. The growth of mobile apps for health-related purposes will continue to increase as mobile devices become more ubiquitous [10].

Despite the number of mobile health apps available, mobile apps used in health care are in their infancy. The majority of mobile health apps researched for mHealth interventions have focused on behavioral changes and self-management. Review of mobile health apps for diabetes, smoking cessation, asthma, and sexually transmitted disease and HIV prevention indicate that most of the available apps do not follow established guidelines; these apps may have questionable reliability, may lack comprehensive information, and may not be personalized [11-14]. Additionally, mobile health apps are often developed without the end user (ie, the patient or health care provider) in mind [8,15-17]. It is also unclear whether or not patients would be interested in a mobile health app as a means for a health care service. Regarding a mobile app for patient education, the objectives of this study were to (1) quantify patient interest, (2) determine desirable features, and (3) determine if a relationship exists between patient variables and interest in an iPad app for patient education.

## Methods

### Setting

The three study sites offered clinical pharmacy services in the Chicago metropolitan area and included two suburban outpatient sites where outpatient ambulatory care services (OACS) are provided and one urban hospital site where hospital-based ambulatory care transition services (HATS) are provided. At the OACS sites, clinical pharmacists provide face-to-face medication therapy management to patients who have been referred by their primary care provider. The OACS sites were comprised of one private internal medicine physician group and one primary care office associated with a patient-centered medical home. At the HATS site, clinical pharmacists provide medication reconciliation and counseling to patients at discharge from the hospital. These patients are being discharged from inpatient treatment on a cardiac medicine floor or one of three internal medicine floors.

Patients were recruited by a sample of convenience. Patients under the age of 18 or for whom English is not their primary language were excluded from the study. Return of the completed survey indicated consent.

### Survey Instrument

A paper-based questionnaire was developed and administered to consenting patients receiving care within the three predetermined sites. The survey was comprised of 19 multiple-choice questions and one open-ended question for general comments. The multiple-choice component included eight questions related to background demographic information, five questions related to technology use, and six questions related to interest in iPad apps and app features. Two of the questions related to technology were only to be answered by respondents who indicated that they own or proficiently use a tablet device or mobile phone. Some questions allowed respondents to “check all that apply.”

To ensure a standardized baseline level of knowledge, a laminated information card titled “What is an iPad?” was provided to the patient after the patient’s knowledge of an iPad was assessed. The iPad was chosen as a platform identifier that the general public was thought to be familiar with at the time the survey was conducted.

The survey was pilot-tested by a group of pharmacy faculty, refined, then pilot-tested by patients prior to distribution.

### Statistical Analysis

A total of 120 surveys were to be administered, split evenly between OACS and HATS, in order to investigate the relationship between certain patient demographics and an interest in an iPad app. The total number of surveys was estimated based on collecting approximately 10 surveys per variable of interest.

The authors identified 12 variables of interest: diabetes, heart failure, asthma/chronic obstructive pulmonary disease (COPD), history of venous thromboembolism (VTE), perceived level of health, knowledge of what an iPad is, types of technology used, age, sex, race/ethnicity, level of education, and type of health insurance. Any answer that was left blank by the survey respondent was not included in the analysis.

Variables of interest were collapsed into categories and analyzed as follows: presence or absence of each of the chronic disease states (ie, diabetes, heart failure, history of VTE, asthma/COPD); perceived level of health (ie, positive—excellent, very good, good—or negative—fair, poor, very poor); knowledge of what an iPad is (ie, yes or no); use of technology (ie, computer, game consoles, mobile phone, tablet device, other portable electronic device, none); age (ie, <40, 40-49, 50-59, 60-69, 70-79, >79); sex (ie, male, female, other); race/ethnicity (ie, American Indian or Alaskan Native, Asian/Pacific Islander, black/African American, Hispanic American, white/Caucasian, other); level of education (ie, high school or less, some college or associate degree, bachelor’s degree or higher); and type of health insurance (ie, Medicare/Medicaid, other public, private [employer sponsored], private [individually purchased], self pay). Descriptive statistics for all parameters were reported.

Ad hoc analyses of variables included number of chronic disease states (ie, 1, 2, 3, 4+, 1+); number of technology devices owned (ie, 1, 2, 3, 4, 5, 3+); age (ie, <50, 50-70, >70); race/ethnicity (ie, white, nonwhite); and type of health insurance (ie, government sponsored, private, self-pay).

This study was designated exempt by the Midwestern University Institutional Review Board.

## Results

Survey collection was complete with 121 collected surveys. Data collection began in December 2012 and ended in July 2013.

### Demographics and Background Information

The mean age of all respondents was 63.7 years and the majority of respondents were between 50 and 70 years old. Less than half of the respondents identified as male (56/120, 46.7%). The most predominant self-identified race/ethnicity was white/Caucasian (94/120, 78.3%), followed by black/African American (11/120, 9.2%). Of the four chronic health conditions identified as variables of interest, diabetes was the most common (43/120, 35.8%), followed by heart failure (27/120, 22.5%), history of VTE (21/120, 17.5%), and asthma/COPD (17/120, 14.2%). Most of the respondents owned at least one technology device and knew what an iPad was.

OACS patients were more likely to have fewer chronic health conditions, a more positive perception of health, and a history of VTE. They were also more likely to identify as white/Caucasian (52/60, 87%) and male (32/60, 53%). HATS patients were more likely to have asthma/COPD, have completed a high school diploma or less, and be racially/ethnically diverse. Additional demographics of the survey respondents are outlined in Table 1.

### Interest in mHealth Apps

Overall interest in a mobile health app for patient education was 63.7% (72/113). Interest increased to 68.4% (78/114) if a health care provider recommended it. Compared to the general study population, HATS patients were slightly more interested on their own accord (68%) and 75% of this population would use a mobile app if a health care provider recommended it. Regardless of the variable of interest and population, in most cases, the respondent’s interest in a mobile app increased if a health care provider recommended it (see Table 2).

Based on number of chronic health conditions, respondents with one condition were most likely to be interested in an app. More than 60% of patients with at least one disease state of interest were interested in using an app. Respondents with a history of VTE were mostly likely to be interested in using an app on their own accord, while respondents with diabetes were mostly likely to be interested if their health care provider recommended it. As the respondent’s perception of their health decreased, their interest in an app lessened.

Respondents who knew what an iPad was were more likely to be interested in using an app (73.1% vs 45.5%). Number of technology devices owned was directly proportional to interest in a mobile app. Of respondents who owned three or more devices, 95.8% would use a mobile app if their health care provider recommended it.

Compared to those aged <50 and >70 years, respondents who were 50-70 years old were most likely to be interested in a mobile app. The two age groups with the highest interest in apps were 60-69 years old (86.5%) and <40 years old (83.3%). No notable differences in interest were identified based on the respondent’s sex. Respondents who identified as white were less likely to be interested compared to nonwhite respondents (60.9% vs 77.3%) and were less likely to use the app if it was recommended by a health care provider (65.5% vs 86.4%). A vast majority of patients with employer-sponsored or privately purchased insurance was interested in an app (78.4%) and would use it if their health care provider recommended it (81.1%).

Many of the same patterns in the overall population were consistent within the OACS and HATS populations. However, OACS respondents <50 years old were slightly more interested than were respondents 50-70 years old and those >70 years old. OACS respondents with a negative perception of health were more likely to be interested in an app, while the opposite was true for HATS patients. Respondents within the HATS populations who had one or two chronic conditions had a very high likelihood (>80%) of using an app if recommended by a health care provider.

**Table 1:** Interest in mHealth app stratified by demographic and clinical characteristics of 120 respondents.

Variable		Interested in an app, n (%)	Interested in an app if recommended by health care professional, n (%)
**Number of diseases**			
	One (n=28)	19 (68)	21 (75)
	Two (n=27)	18 (67)	15 (56)
	Three (n=32)	20 (63)	23 (72)
	Four or more (n=55)	35 (64)	40 (73)
	One or more (n=109)	71 (65.1)	75 (68.8)
**Chronic disease states**			
	Diabetes (n=40)	27 (68)	32 (80)
	Heart failure (n=23)	16 (70)	14 (61)
	History of VTE^a^ (n=20)	15 (75)	14 (70)
	Asthma/COPD^b^ (n=22)	13 (59)	14 (64)
**Perceived level of health**			
	Positive (n=77)	51 (66)	53 (69)
	Negative (n=34)	20 (59)	23 (68)
**Knowledge of an iPad**			
	Yes (n=78)	57 (73)	61 (78)
	No (n=33)	15 (45)	16 (48)
**Technology**			
	No devices (n=20)	8 (40)	7 (35)
	One device (n=39)	22 (56)	23 (59)
	Two devices (n=24)	20 (83)	21 (88)
	Three or more devices (n=24)	20 (83)	23 (96)
**Sex**			
	Male (n=54)	35 (65)	36(67)
	Female (n=57)	37 (65)	41(72)
**Age in years**			
	<50 (n=18)	13 (72)	14 (78)
	50-70 (n=51)	39 (76)	14 (86)
	>70 (n=40)	19 (48)	17 (43)
**Race**			
	White (n=87)	53 (61)	57 (66)
	Nonwhite (n=22)	17 (77)	19 (86)
**Education**			
	High school diploma or less (n=32)	18 (56)	18 (56)
	Associate degree or some secondary education coursework (n=42)	30 (71)	33 (79)
	Bachelor’s degree or more (n=34)	23 (68)	24 (71)
**Type of insurance**			
	Government sponsored (n=64)	37 (58)	41 (64)
	Employer sponsored or purchased (n=37)	29 (78)	30 (81)
	Self-pay (n=8)	4 (50)	4 (50)

^a^VTE: venous thromboembolism.

^b^COPD: chronic obstructive pulmonary disease.

**Table 2:** Patient interest in a mobile app for patient education in pharmacy practice increased if recommended by a health care professional.

Population variable	Interested in an app, n (%)	Interested in an app if recommended by health care professional, n (%)
All respondents	72/113 (63.7)	78/114 (68.4)
HATS^a^ (n=57)	39 (68)	43 (75)
OACS^b^ (n=55)	33 (60)	34 (62)

^a^HATS: hospital-based ambulatory care transition services.

^b^OACS: outpatient ambulatory care services.

**Table 3:** Desirable methods for using a mobile health app for patient education (n=109).

Options	n (%)
I want my health care provider to use and demonstrate the app	37 (33.9)
I want to have access to the app at home	58 (53.2)
Both	22 (20.2)

### Technology Use Preferences

A majority of respondents (81.3%) owned at least one device, which was always a computer. Of those that owned two devices, the second device was almost always a mobile phone; of those that owned three devices, the most common combination was computer, mobile phone, and tablet. Mobile phone and tablet users indicated that they use their devices at least daily (86.2%). The most common apps used were weather apps, search tool apps, and news apps. Health and fitness apps were used by 30.9% of mobile device owners.

When asked how they prefer to receive information about their health, 5.7% of respondents indicated software apps. Less than half the respondents have seen an iPad used in a health care setting.

Regarding methods for using a mobile health app, the majority of respondents were more interested in having access to the app at home (58/109, 53.2%) compared to having the health care provider use and demonstrate the app (37/109, 33.9%); some patients would prefer both (22/109, 20.2%). Table 3 indicates the distribution of responses.

Respondents indicated an interest in a variety of health-related information, with the most common being information about the medications they are taking (see Table 4). The two most common methods to display information were articles with printed text (56/95, 59%) and images and diagrams (46/95, 48%), while the least common were podcasts and audio recordings (20/95, 21%).

**Table 4:** Types of information and methods to display information that patients would find beneficial in a mobile health app for patient education.

Information and methods of display		n (%)
**Type of information (n=105)**		
	Medications	87 (82.9)
	Health conditions	81 (77.1)
	Lifestyle changes	58 (55.2)
	Treatment options	68 (64.8)
	Tools and resources to improve health	59 (56.2)
**Methods to display information (n=95)**		
	Articles with printed text	56 (59)
	Images and diagrams	46 (48)
	Links to resources	41 (43)
	Interactive tutorials	39 (41)
	On-demand educational videos	27 (39)
	Animation with narration	28 (30)
	Podcasts or audio recordings	20 (21)

**Figure 1:**
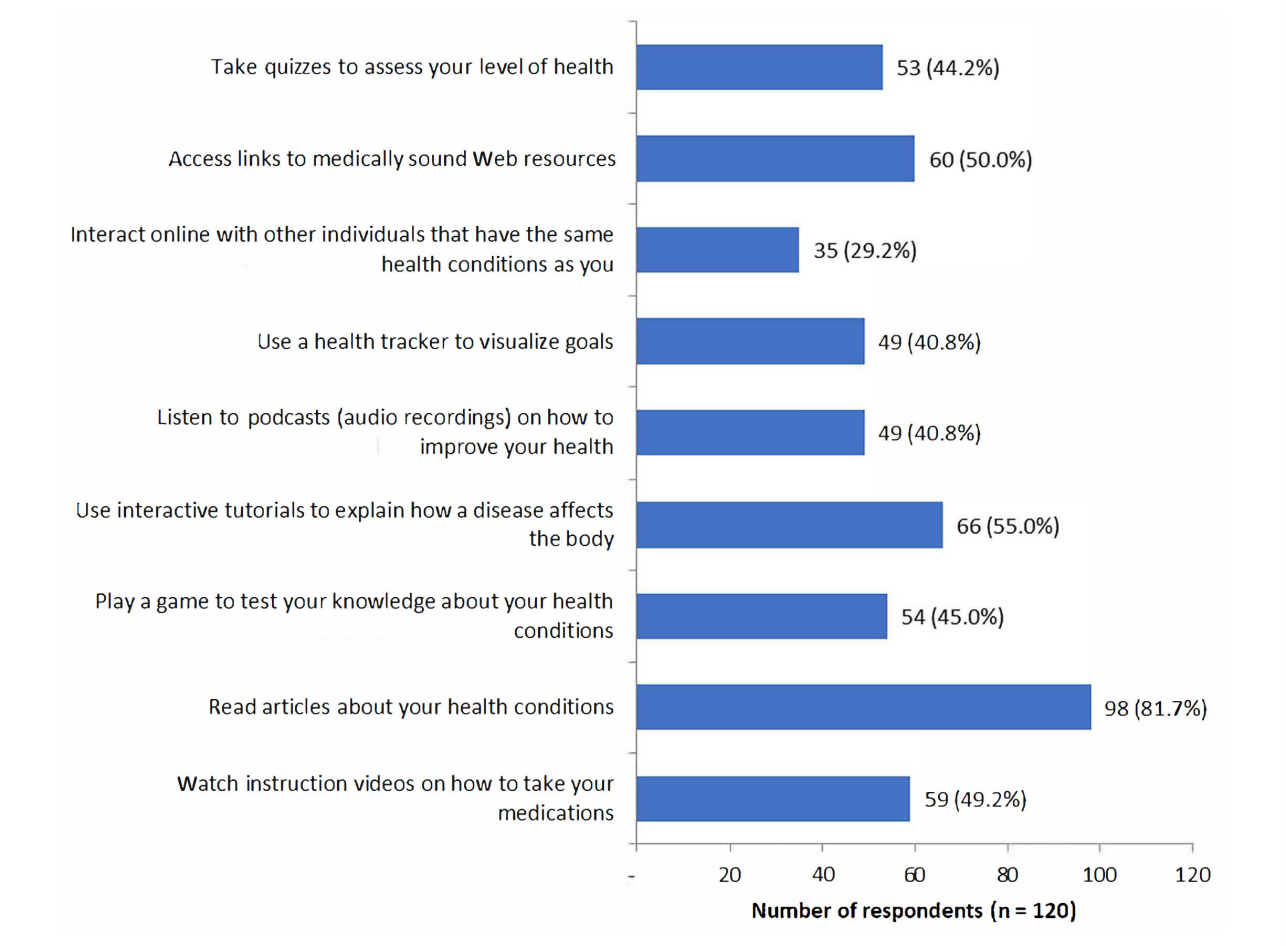
Actions respondents would be likely or very likely to take when using a mobile health app for patient education.

Respondents were also asked to identify their likelihood of performing various actions related to patient education as shown in Figure 1. Relevant comments written in response to the open-ended question included one comment asking for both Kindle and Blackberry software to be included.

## Discussion

### Principal Findings

Overall, a majority of respondents indicated that they were interested in a mobile app for patient education. Almost all patient variables indicated a large interest in an app. Interested respondents were younger, had some amount of secondary education, owned one or more devices, received private health care coverage, and had basic knowledge of an iPad. A digital divide grew as interest decreased dramatically for those over age 70, for those that do not own any mobile technology devices, and for those who pay for health care fully out of pocket (ie, self-pay).

Survey respondents were more likely to be interested in an app they could use at home versus a provider-driven, point-of-care app. A similar percentage of those who reported seeing a health care provider use an iPad were interested in an app at the point of care. This interest may increase as exposure to mobile technology increases. Concurrently, there may be an increased expectation from patients for health care providers to use mobile apps when providing care to patients.

If a health care provider recommended an app, there was an almost universal increase in interest. Power, size, portability, built-in features, access to the Internet, social and communication tools, personal affection, and a large marketplace makes using a mobile app for patient education attractive to patients [9]. These factors increase a health care professional’s abilities to provide point-of-care or just-in-time services to patients, both in practice settings and remotely. This would also support the notion of app prescribing.

A potential starting place for health care providers to introduce mHealth apps into patient care is recommending specific apps to patients that they can access on their own device at any time. Health care providers may choose to target certain populations, such as younger patients with one chronic health condition and a positive perception of health, in deciding to whom they should recommend mHealth apps. Apps may not be best suited for patients with a preference for personal consultation from their provider, approximately a third of patients over 55 [18].

It is interesting to note that the perception of health influenced an interest in app technology in different ways based on the type of health care setting of the respondent. Ambulatory patients in primary care settings were more likely to be interested if they had a negative perception of their health. These individuals with poor health and many chronic conditions are usually the most vulnerable and tend to require extensive patient education. On the other hand, patients who were about to be discharged from the hospital, who had a positive perception of health and only one chronic condition, were more likely to be interested in mobile apps for health-related information. Discharge counseling at this point in time is crucial for many patients because they are likely to be more receptive to information to promote a healthy lifestyle, prevent disease progression, and avoid readmission. Utilization of apps among these types of patients at discharge from the hospital may be beneficial.

When asked about what types of information they would find beneficial, respondents indicated that they want more information in a wide variety of areas and formats. We believe that this suggests that options for app developers are bountiful. Interestingly, respondents indicated most frequently that they are interested in articles, printed text, images, and diagrams as delivery methods for medical information. Use of these delivery methods for educating patients has been common practice for years. We wonder if this was due to familiarity and, furthermore, wonder if many of the respondents knew what podcasts and audio recordings are. With more education as to what podcasts and audio recordings are, patients may potentially be more interested in these as delivery methods within mobile apps for health-related information.

### Limitations

The focus of the survey specifically on the iPad device may limit our ability to generalize this data to other mobile technology devices and mHealth apps. Additionally, the survey was conducted early in the adoption of the tablet devices. We chose to focus solely on the iPad as investigators felt it was the most recognizable mobile device on the market; inclusion of other devices could have confused patients, especially those that were not familiar with tablet devices and would have required an explanation of other tablet devices. Respondents who may be interested in receiving education through the use of other devices may not have identified an interest in the use of the iPad. There were several comments to this effect that were relayed verbally to the survey administrator, who is the primary author. We suspect that interest in the use of an iPad, or any tablet device, would not have decreased from the time that this study was conducted.

Of the surveyed population, approximately one-third did not know what an iPad was. For some of these respondents, the laminated information card could have provided enough information to trigger the respondent’s memory. For other respondents that may not have ever encountered an iPad, it is possible that some of them may not have been able to conceptualize the device, even despite reading the description and viewing the life-size picture. Demonstrating the use of an iPad device and/or app to respondents was assessed during survey development, but ultimately was not utilized due to feasibility.

One question in particular was designed to provide app developers with more insight into what patients want in an app for patient education, as this has been suggested to be an important aspect of the design process [16]. Unfortunately, there was a low response rate for this question. The low response rate was likely due to the fact that the question was asked in a matrix and patients did not understand the format. With the data that we have, we are able to suggest some areas for app development based on the likelihood that patients would use certain features, but future research in this area is warranted.

The patient populations of each site are uniquely different and cover a broad range of demographics; therefore, inherent bias exists. Additionally, the study utilized a sample of convenience, as patients were not randomly selected. OACS patients report to clinic for appointments with clinical pharmacists. Those who adhere to appointments may be more likely to want additional education, though this survey was not conducted in a manner to assess this thought. Patients who did not show up for appointments were not able to complete a survey. Thus, the population of patients surveyed may not represent the entirety of the OACS patient population.

The population of survey respondents at HATS may have included some patients who were not actually discharged from the hospital setting. We made every attempt to capture patients that were ambulant and had a high likelihood of being discharged from the hospital. However, the clinical status changed for some of the patients and they continued to be hospitalized after survey completion. Surveys were deidentified and we were not able to exclude patients who were not discharged. It is notable that being hospitalized was not specifically addressed as an exclusion criterion for this study. Including surveys from a broad patient population in different health care settings may affect the homogeneity of the respondents, but it may also strengthen the results by reflecting the diverse opinion(s) patients have.

### Strengths and Future Research

One of the strengths of this study is that it included patients who previously did not know what an iPad was. Despite this lack of prior knowledge, many of these respondents indicated an interest in obtaining health-related information via mobile apps. This counters a preconceived notion that those who are older and may not have used mobile devices or know what one is will be less likely to want to learn in this way.

Additionally, the responses suggested that patients would use apps for education if their health care providers recommended them. Given this data, providers should be guiding patients to resources. Health care professionals in primary care and transition-of-care settings should carefully weigh the pros and cons of utilizing this technology with their patients.

Despite patient interest and known benefits of patient education for chronic disease, not much is known regarding the utility of mHealth apps for patient education. Published studies assessing the impact of mHealth apps on clinically relevant health outcomes are limited. However, early data show promise [19-21].

Future areas of research include looking further into the desirable features of apps for patient education. Investigators and developers need to include patients and health care providers in this area of research. With more data in this area, it will be possible to develop and utilize an app for educational interventions that is tailored to a patient and/or a health care provider and that impacts health outcomes.

### Conclusions

Interest in an mHealth app for patient education is high, regardless of patient variables. A health care provider’s recommendation may increase the likelihood of a patient using an app. This emphasizes the need to develop evidence-based, medically sound, and reliable mobile apps that both patients and pharmacists can use and trust.

## Abbreviations

COPD: chronic obstructive pulmonary disease
HATS: hospital-based ambulatory care transition services
OACS: outpatient ambulatory care services
PDA: personal digital assistant
VTE: venous thromboembolism
